# A simulated maximum likelihood procedure for analyzing imprecise trade‐off thresholds between the benefits and harms of medicines

**DOI:** 10.1002/sim.9583

**Published:** 2022-09-26

**Authors:** Douwe Postmus, Francesco Pignatti, Hans L. Hillege, Tommi Tervonen

**Affiliations:** ^1^ Department of Epidemiology University of Groningen, University Medical Center Groningen Groningen The Netherlands; ^2^ Oncology & Haematology Office Amsterdam The Netherlands; ^3^ Kielo Research Zug Switzerland

**Keywords:** additive value function, benefit‐harm assessment, patient preferences, simulated maximum likelihood, thresholding

## Abstract

Stated preference studies in which information on the willingness to trade‐off between the benefits and harms of medicines is elicited from patients or other stakeholders are becoming increasingly mainstream. Such trade‐offs can mathematically be represented by a weighted additive function, with the weights, whose ratios determine how much an individual is willing to trade‐off between the treatment attributes, being the response vector for the statistical analysis. One way of eliciting trade‐off information is through multi‐dimensional thresholding (MDT), which is a bisection‐based approach that results in increasingly tight bounds on the values of the weights ratios. While MDT is cognitively less demanding than other, more direct elicitation methods, its use complicates the statistical analysis as it results in weights data that are region censored. In this article, we present a simulated maximum likelihood (SML) procedure for fitting a Dirichlet population model directly to the region‐censored weights data and perform a series of computational experiments to compare the proposed SML procedure to a naive approach in which a Dirichlet distribution is fitted to the centroids of the weights boundaries obtained with MDT. The results indicate that the SML procedure consistently outperformed the centroid‐based approach, with the centroid‐based approach requiring three bisection steps per trade‐off to achieve a similar precision as the SML procedure with one bisection step per trade‐off. Using the newly proposed SML procedure, MDT can be applied with smaller sample sizes or with fewer questions compared to the more naïve centroid‐based approach that was applied in previous applications of MDT.

## 1 INTRODUCTION

There has been an increased interest in exploring ways to incorporate patient preference information in benefit‐harm decision making throughout a medicine's lifecycle.^1^ These include situations in regulatory decision making where the treatment benefit‐harm balance is not self‐evident and patient preference information may be used to identify subgroups of patients who would be willing to tolerate treatment harms for obtaining the benefits.^2^

Different theoretical models to describe treatment preferences have been proposed in the literature.^3^ In this article, we focus on preference models that are grounded in the economic theory of rational choice, which states that individuals make choices in a way that maximizes their utility. Under this assumption, the preference ordering of the available treatments can be represented by a real‐valued function, which is usually being referred to as a value function. The parameters of this function vary across individuals depending on the rate at which they are willing to trade‐off among the attributes that characterize the treatments. Estimating how the preference model parameters are distributed in the population and exploring to what extent these distributions vary between subgroups of patients, defined based on demographic and clinical characteristics, are often main objectives of a stated preference study.

Different strategies exist for eliciting and analysing treatment preferences in the context of an additive value function.^3^ This article focuses on multi‐dimensional thresholding (MDT), which can be seen as a multi‐dimensional version of the thresholding technique.^4^ Compared to other, cognitively more demanding, elicitation methods such as swing weighting,^5^ MDT does not result in exact values of the attribute weights. Instead, what is known at the end of an MDT exercise is that a subject's weight vector is bound to lie within a certain subspace of the set of all possible weight vectors, resulting in response variables that are region censored.

In previous applications of MDT,^6^, ^7^ the statistical analysis was performed on the centroids of the subspaces containing the subjects' true weight vectors, thereby disregarding any uncertainty stemming from the unknown true distribution of the data within these subspaces. This centroid‐based approach is therefore likely to result in biased and inefficient estimates, especially in practical applications of MDT where the number of bisection questions is constrained to a reasonable number to limit respondent burden. The purpose of this article is to introduce a more advanced simulated maximum likelihood (SML) procedure for fitting a Dirichlet population model directly to the region‐censored MDT data as well as to present the results of a series of computational experiments in which the SML procedure is compared against the previously applied centroid‐based approach.

## 2 ILLUSTRATIVE CASE STUDY

To illustrate our approach, we use the data from a previous study in which stated preferences for hypothetical cancer treatments were elicited from 560 patients with multiple myeloma.^7^ The beneficial effects of treatment were measured in terms of the attribute *one‐year progression‐free survival* (PFS), ranging from 50% to 90%. The harmful effects of treatment were measured in terms of the attributes *risk of moderate but chronic toxicity* (MT), ranging from 45% to 85%, and *risk of severe toxicity* (ST), ranging from 20% to 80%. With these attributes and scale ranges, each treatment under consideration can be characterized through its attribute vector $x=(x_{\mathrm{PFS}},x_{\mathrm{MT}},$ $x_{\mathrm{ST}})$, with the attribute values $x_{\mathrm{PFS}},x_{\mathrm{MT}}$, and $x_{\mathrm{ST}}$ being bounded from below and above by the scale ranges defined for these attributes.

Assuming that the conditions for applying the additive value function are met,^8^ the utility that a respondent obtains from a treatment with attribute vector $x=(x_{\mathrm{PFS}},x_{\mathrm{MT}},$ $x_{\mathrm{ST}})$ can be written as

(1) $$v\left(x_{\mathrm{PFS}},x_{\mathrm{MT}},x_{\mathrm{ST}}\right)=w_{\mathrm{PFS}}\cdot v_{\mathrm{PFS}}\left(x_{\mathrm{PFS}}\right)+w_{\mathrm{MT}}\cdot v_{\mathrm{MT}}\left(x_{\mathrm{MT}}\right)+w_{\mathrm{ST}}\cdot v_{\mathrm{ST}}\left(x_{\mathrm{ST}}\right),$$

where $v_{\mathrm{PFS}},v_{\mathrm{MT}}$, and $v_{\mathrm{ST}}$ are one‐dimensional functions that transform the attribute values from their original measurement scales to a 0 to 1 interval scale of increasing preference, and $w_{\mathrm{PFS}}$, $w_{\mathrm{MT}}$, and $w_{\mathrm{ST}}$ are weights that vary across individuals depending on their willingness to trade‐off between the three attributes. Because only ratios of weights have meaning ‐ for example, $\frac{w_{\mathrm{PFS}}}{w_{\mathrm{ST}}\;}=2$ implies that increasing 1‐year PFS from 50% to 90% is considered to be twice as important as decreasing the risk of severe toxicity from 80% to 20% ‐ the weights are normalized to sum up to a given constant. The convention is to use 1 as the normalization constant, so that the set of all possible weight vectors coincides with the unit simplex $∆=\left\{\left(\;w_{\mathrm{PFS}},w_{\mathrm{MT}},w_{\mathrm{ST}}\right)\in R^{3}\;\right|\;w_{\mathrm{PFS}}+w_{\mathrm{MT}}+w_{\mathrm{ST}}=1,\;\;w_{\mathrm{PFS}},w_{\mathrm{MT}},w_{\mathrm{ST}}\geq 0\}$, which can geometrically be represented as an equilateral triangle with vertices (0, 0, 1), (0, 1, 0), and (0, 0, 1).

The partial value functions $v_{\mathrm{PFS}},v_{\mathrm{MT}}$, and $v_{\mathrm{ST}}$ can either assumed to be linear, which implies that the marginal rate of substitution between the treatment attributes is constant, or elicited from study participants, which may be more appropriate in situations where diminishing or increasing marginal utilities are expected. Here, we shall assume, without loss of generalizability, that all partial value functions are linear, so that the weights remain the only unknown preference model parameters.

## 3 THE MDT METHOD

### 3.1 General description of the MDT method

MDT is a questioning procedure for eliciting the weights of an additive value function over treatment attributes measured on ordinal or numeric scales, such as changes from baseline in a biomarker or risk of side effects. It consists of an initial ranking phase in which an ordinal ranking of the attribute weights is obtained, followed by a thresholding phase in which bounds on the weights ratios of consecutively ranked attributes are elicited through repeated application of the thresholding technique.^4^

An ordinal ranking of the attribute weights can be efficiently obtained through a swing ranking procedure.^8^ Swing ranking works by starting with a reference alternative that performs worst on all attributes. Respondents are then asked to select the attribute that they would foremost want to improve from the worst to the best value. Following their initial selection, the reference alternative is updated with the chosen attribute at its best value after which the questioning procedure is repeated until all attributes are at their best value. In this way, a full ranking of the attribute weights is obtained after *n‐1* elicitation questions, where *n* is the number of attributes under consideration.

Thresholding involves asking respondents to consider two hypothetical treatments that present a trade‐off between a pair of attributes *i* and *j*, labeled such that the improvement from worst to best on attribute *i* is preferred to the improvement from worst to best on attribute *j*. The treatment with the worst possible value for attribute *i* and the best possible value for attribute *j* is selected as the reference alternative. The treatment with the worst possible value for attribute *j* and a value $x_{i}$ between worst and best for attribute *i* is used as the target alternative. The threshold value is the value of $x_{i}$ that makes the respondent indifferent between the reference alternative and the target alternative. Rather than asking the respondent to provide the value of $x_{i}$ directly (direct elicitation), the thresholding technique homes in on this threshold value through a series of pairwise comparison questions (indirect elicitation). After a set number of bisection steps, the questioning procedure is terminated, resulting in a lower‐ and upper‐bound on the ratio of the weights $\frac{w_{i}}{w_{j}}$ corresponding to that trade‐off. In MDT, the thresholding procedure is repeated until all *n‐1* trade‐offs between the consecutively ranked attributes have been evaluated or until a maximum number of pairwise comparison questions has been reached.

### 3.2 Illustration of MDT in the context of our example problem

**FIGURE 1:**
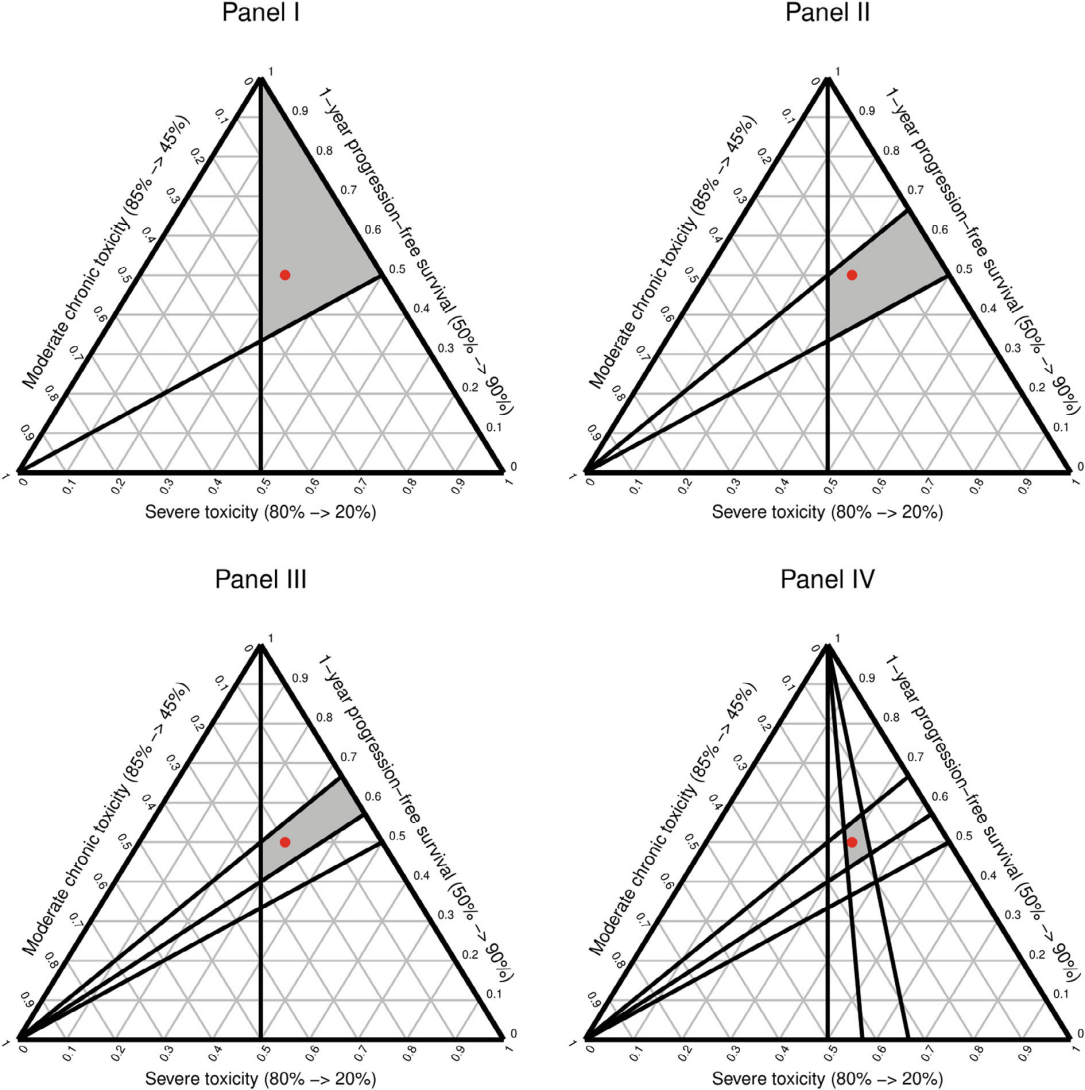
Example of the steps in the MDT procedure. Panel I (top left): feasible weight space after the ordinal ranking of the attribute scale swings, Panel II (top right): feasible weight space after the first pairwise comparison question for the trade‐off between 1‐year progression‐free survival and severe toxicity, Panel III (bottom left): feasible weight space after the second pairwise comparison question for the trade‐off between 1‐year progression‐free survival and severe toxicity, Panel IV (bottom right): feasible weight space at the end of the MDT procedure. The red point in each panel represents the respondent's “true” weight vector

To illustrate the working of MDT in the context of our example problem with two bisection steps per trade‐off, consider a respondent with the weight vector $\left(w_{\mathrm{PFS}},w_{\mathrm{MT}},w_{\mathrm{ST}}\right)=\left(0.5,0.2,0.3\right)$. At the start of the MDT procedure, the feasible weight space, that is, the set of weight vectors that are consistent with the preference information provided by the respondent, is equal to the full unit simplex ∆. After the initial ranking phase, the feasible weight space reduces to the subspace Ω⊆∆ that contains all weight vectors for which $w_{\mathrm{PFS}}>w_{\mathrm{ST}}>w_{\mathrm{MT}}$. This subspace corresponds to the grey area shown in Figure 1, panel I. In the next step of MDT, the thresholding technique is applied to establish the trade‐offs the respondent is willing to accept between 1‐year PFS and the risk of ST. In the first pairwise comparison question, the respondent is asked to choose between the reference alternative (50% PFS, 45% MT, 20% ST) and the target alternative (70% PFS, 45% MT, 80% ST), where 70% PFS is the midpoint of the attribute scale for PFS. With linear partial value functions, this choice question can geometrically be represented by the hyperplane $\frac{w_{\mathrm{PFS}}}{w_{\mathrm{ST}}}=2$ that cuts the simplex from the bottom left vertex to the point $\left(w_{\mathrm{PFS}},w_{\mathrm{MT}},w_{\mathrm{ST}}\right)=\left(2/3,0,1/3\right)$ on the edge formed by the other two vertices (Figure 1, panel II). This hyperplane divides the simplex into an upper half‐space $\frac{w_{\mathrm{PFS}}}{w_{\mathrm{ST}}}\geq 2$ containing all weight vectors resulting in a preference for the target alternative and a lower half‐space $\frac{w_{\mathrm{PFS}}}{w_{\mathrm{ST}}}\leq 2$ containing all weight vectors resulting in a preference for the reference alternative. The weight vector of our example respondent lies somewhere in this lower half‐space, so when faced with the presented choice question, they will choose the reference alternative. In the second pairwise comparison question, the respondent is asked to choose between the reference alternative (50% PFS, 45% MT, 20% ST) and the target alternative (80% PFS, 45% MT, 80% ST), where 80% PFS is the midpoint of the upper half of the attribute scale for PFS. This question can be represented by the hyperplane $\frac{w_{\mathrm{PFS}}}{w_{\mathrm{ST}}}=4/3$. This time, the respondent's weight vector lies somewhere in the upper half‐space $\frac{w_{\mathrm{PFS}}}{w_{\mathrm{ST}}}\geq 4/3$, so they will select the target alternative. The updated feasible weight space $\;\Omega^{\prime }\subseteq \Omega$ after these two bisection steps is represented by the grey area in Figure 1, panel III. In the final step of the MDT procedure, the thresholding technique is applied to establish the trade‐offs the respondent is willing to accept between the risks of ST and MT. The grey area in Figure 1, panel IV shows the feasible weight space for our example respondent at the end of the MDT procedure.

## 4 STATISTICAL ANALYSIS OF MDT DATA

MDT leads, for each subject, to a set of feasible weights. Previous works on MDT^6^, ^7^, ^9^ subsequently determined the centroids of these feasible weight spaces to obtain a single representative weight vector for each subject. However, performing the statistical analysis on the centroids of the feasible weight spaces may result in biased estimates when using the data collected in an MDT exercise to estimate the population distribution of the attribute weight vectors. In this section, we first describe how SML estimation can be applied to estimate the parameters of this distribution directly from the region‐censored data, after which we apply the approach to the MDT data collected in the multiple myeloma case study.

### 4.1 General description of the proposed SML procedure

Let the random vector $W_{j}$ be the weight vector of the *j*th subject and let $\Omega_{j}$ be the set of all possible weight vectors consistent with the preference information elicited from that subject. The probability that $W_{j}$ is contained within $\Omega_{j}$ is

(2) $$P_{j}=\int_{\mathrm{w\epsilon}\Omega_{j}}f\left(w\right)\mathrm{dw},$$

where f(w) is the density of $W_{j}.$ Throughout this article, it is assumed that the individual weight vectors are independent and identically Dirichlet distributed, so that $f\left(w\right)=\frac{1}{B\left(\alpha \right)}\prod_{i=1}^{n}{w_{i}}^{\left(\alpha_{i}-1\right)}$, where $\alpha =\left(\alpha_{1},\ldots ,\alpha_{n}\right)$ is the parameter vector of the Dirichlet distribution, B(α) the multinomial beta function, and *n* the number of attributes under consideration. Using the mean‐precision parametrization of the Dirichlet distribution,^10^ the parameter vector α can be written as the product of the distribution's mean $\mu =\left(\mu_{1},\ldots ,\mu_{n}\right)$ and a precision parameter ϕ that is inversely related to the distribution's variance. With this notation, the log‐likelihood function can be expressed as

(3) $$\mathrm{LL}\left(\mu ,\varphi \right)=\sum_{j=1}^{N}\log P_{j},$$

where *N* is the number of respondents.

Maximizing the log‐likelihood function requires the evaluation of the integrals in (2). Because these integrals cannot be evaluated analytically, they need to be approximated numerically through simulation. An intuitive approach to estimate $P_{j}$ is to sample a large number of weights from f(w) and to then calculate the proportion of these weights that are contained within $\Omega_{j}$. However, because the ratio of the volume of $\Omega_{j}$ to the volume of the full unit simplex is very small, this form of accept‐reject sampling is not practically feasible.

The computational difficulties associated with trying to hit a small region with random samples drawn from a much larger domain can be avoided by sampling directly from $\Omega_{j}$. Uniform samples from this convex polytope can be efficiently obtained by applying previously proposed sampling algorithms, such as hit‐and‐run sampling.^11^, ^12^ The density of the uniform distribution over $\Omega_{j}$ is

(4) $$g_{j}\left(w\right)=\frac{1}{k_{j}}\;\left(n-1\right)!,$$

where $k_{j}=\frac{\mathrm{vol}\left(\;\Omega_{j}\right)}{\mathrm{vol}\left(\;\text{simplex}\right)}$ is a normalization constant that ensures that $g_{j}\left(w\right)$ integrates to 1. Using this density, $P_{j}$ can be rewritten as

(5) $$P_{j}=\int_{\mathrm{w\epsilon}\Omega_{j}}f\left(w\right)\mathrm{dw}=\int_{\mathrm{w\epsilon}\Omega_{j}}\frac{f\left(w\right)}{g_{j}\left(w\right)}g_{j}\left(w\right)\mathrm{dw}=\int_{\mathrm{w\epsilon}\Omega_{j}}\frac{k_{j}f\left(w\right)}{\left(n-1\right)!}g_{j}\left(w\right)\mathrm{dw}=\frac{k_{j}}{\left(n-1\right)!}E\left[f\left(w\right)\right],$$

where E[f(w)] is the expected value of f(w) with respect to the uniform distribution over $\Omega_{j}$. This expected value can be approximated numerically by uniformly sampling *m* weight vectors ${\tilde{w}}_{1j},\cdots ,{\tilde{w}}_{\mathrm{mj}}$ from $\Omega_{j}$ and then computing the average $\frac{1}{m}\sum_{i=i}^{m}\;f\left({\tilde{w}}_{\mathrm{ij}}\right)$. Using the simulated values of E[f(w)] to estimate $P_{j}$, the simulated log‐likelihood function can be written as

(6) $$\mathrm{SLL}\left(\alpha \right)=\sum_{j=1}^{N}\log P_{j}=\sum_{j=1}^{N}\log\left(\frac{k_{j}}{\left(n-1\right)!}\right)+\sum_{j=1}^{N}\log\left(\frac{1}{m}\sum_{i=i}^{m}\;f\left({\tilde{w}}_{\mathrm{ij}}\right)\right).$$

Because the simulated log likelihood is maximal whenever $\sum_{j=1}^{N}\log\left(\frac{1}{m}\sum_{i=i}^{m}\;f\left({\tilde{w}}_{\mathrm{ij}}\right)\right)$ is maximal, the values of the normalization constants $k_{1},\cdots ,k_{N}$ do not have to be calculated when applying this SML procedure.

### 4.2 Application to the multiple myeloma case study

**FIGURE 2:**
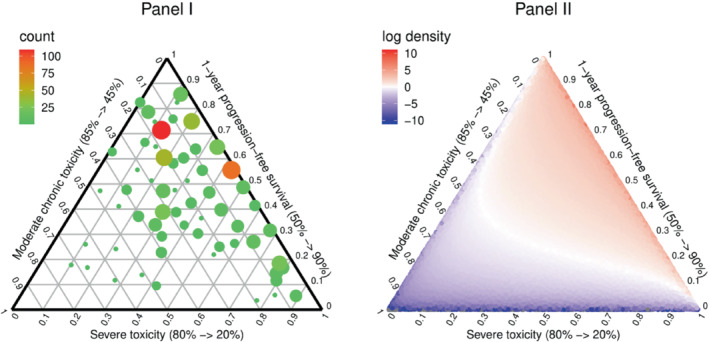
Distribution of the attribute weights in the multiple myeloma case study. Panel I (left) depicts the centroids of the feasible weight spaces elicited from the 560 study participants, with the color and size describing the frequency by which that particular path through the survey was chosen; Panel II (right) shows the log of the Dirichlet density that was fitted to the case study data

In the multiple myeloma case study, MDT was applied with 2 bisection steps per trade‐off after the ranking phase, resulting in 96 possible paths through the exercise's questioning tree. The individual responses of the 560 study participants are summarized graphically in Figure 2, panel I, where each point represents the centroid of the feasible weight space associated with that particular path through the survey. The color and size of each point describes the frequency by which that particular path was chosen. Application of the SML procedure to estimate the parameters of the Dirichlet population model resulted in an estimated mean of (0.53, 0.15, 0.33) and an estimated precision of 4.33. A plot of the log of the density of the fitted Dirichlet distribution is provided in Figure 2, panel II. This plot shows that most of the mass of the fitted distribution is concentrated around the right axis, which suggests that treatment preferences in this patient population are primarily determined by differences in individual trade‐offs between 1‐year PFS and the risk of ST. This is consistent with the descriptive findings in Figure 2, panel I in which there are only a few observations in the neighborhood of the lower‐left corner of the simplex, implying that most study participants attached relatively little importance to the MT attribute during the MDT exercise.

## 5 SIMULATIONS

We conducted simulations to assess the effects of the sample size, the number of bisection steps, the number of attributes, and the estimation procedure on the precision with which the parameters of a set of randomly generated Dirichlet population models for the distribution of the attribute weights were estimated.

Using the mean‐precision parameterization of the Dirichlet distribution, we performed the following two steps to construct those Dirichlet models. First, we randomly sampled the distribution's mean from the uniform distribution over the unit simplex. Second, we sampled the distribution's precision parameter uniformly between 5 and 10, with higher values resulting in distributions that are more heavily concentrated around their mean value. To ensure that the sampled Dirichlet models would still be realistic descriptions of how individual weight vectors might be distributed in practical applications of the MDT procedure, we accepted any distribution for which the ratio of the highest to the lowest element of its mean vector was smaller than 10 and resampled all other distributions. In total, 200 Dirichlet models were generated, evenly split across scenarios with four and six attributes.

In each simulation, we randomly sampled the “true” weight vectors of *N* respondents from the Dirichlet model generated for that simulation, using two different sample sizes: N = 100 and N = 300. These sample sizes were selected based on a previous study that showed that the mean population preference estimates are likely to stay similar with sample sizes larger than 300 in problems with low number of attributes.^9^ For each simulated respondent, we subsequently performed the MDT procedure with a number of bisection steps that was varied between one, two, and three times the number of trade‐offs, yielding a total number of pairwise comparison questions of 3, 6, or 9 for a four attribute problem and 5, 10, or 15 for a six attribute problem (on top of the questions that would be required to complete the initial ranking phase). Finally, we estimated the parameters of the underlying Dirichlet model by using two different estimation approaches: the newly proposed SML procedure and the naïve centroid‐based approach.

For each simulation, the accuracy of the two estimation procedures was assessed by calculating (i) the Euclidean distance from the estimated population mean to the true population mean for that simulation and (ii) the relative difference between the estimated and true value of the precision parameter (ie, [estimated precision − true precision] / true precision). For reference, we also fitted a Dirichlet model directly to the *N* weight vectors used to simulate the MDT responses, reflecting a scenario where exact preference information is elicited and therefore providing an upper bound on the accuracy that can be achieved at a given combination of attributes and sample size.

**FIGURE 3:**
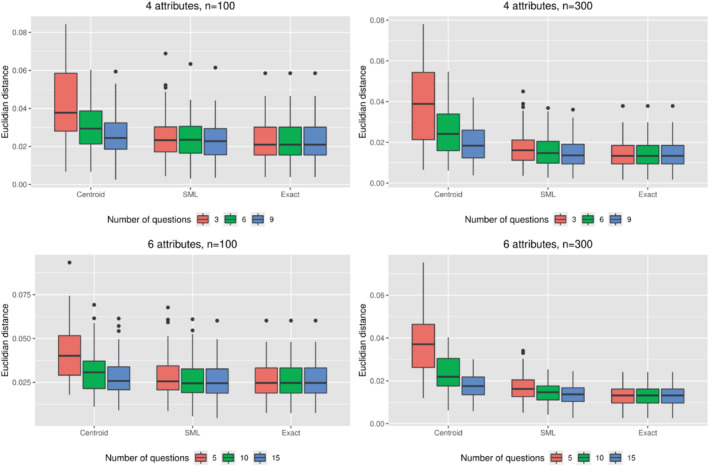
Results of the simulations with four (top) and six attributes (bottom), with sample sizes of 100 (left) and 300 (right) respondents. Boxplots indicate distributions of Euclidean distances from the estimated population mean to the true population mean over 100 simulations. SML, simulated maximum likelihood

**FIGURE 4:**
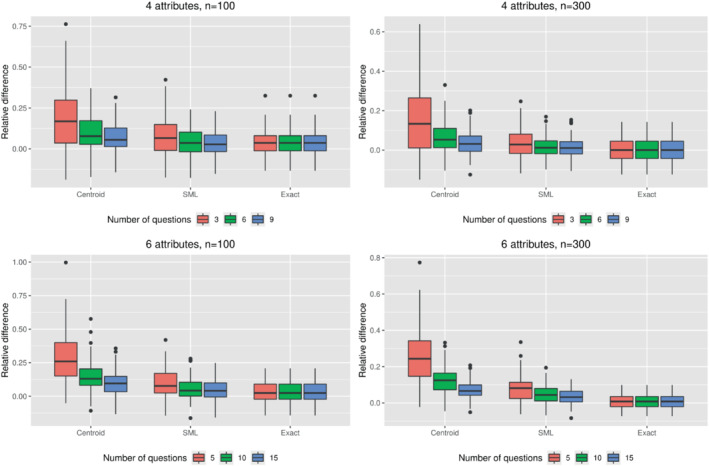
Results of the simulations with four (top) and six attributes (bottom), with sample sizes of 100 (left) and 300 (right) respondents. Boxplots indicate distributions of the relative difference between the estimated and true precision parameter over 100 simulations. SML, simulated maximum likelihood

The results of the simulations are presented in Figures 3 and 4. Figure 3 shows the effect of the estimation procedure on the Euclidean distance between the true and estimated population means as a function of the sample size and the number of bisection steps. By design, the results are unbiased for the exact preference information scenario, meaning that the Euclidean distances for that scenario are a reflection of the sampling variation that is present in the computational experiments. Compared to this reference scenario, the MDT with centroid‐based estimation scenario resulted in considerably higher values for the Euclidean distance, especially for problem instances with 1 bisection step per trade‐off. Because the estimation procedure was the same for these two scenarios (ie, traditional maximum likelihood estimation based on the true or centroid‐imputed weight vectors), the increase in Euclidean distance in the MDT with centroid‐based estimation scenario confirms that the centroid‐based approach results in biased estimates of the distribution mean. When comparing the MDT with SML estimation scenario against the reference scenario, performance is much closer, even in scenarios in which only 1 bisection step per trade‐off was applied. A similar conclusion is reached when looking at the results for the precision parameter (Figure 4), which shows that (i) the centroid‐based approach results in an overestimation of the precision parameter and (ii) that this bias can be considerably reduced by switching from centroid‐based estimation to SML estimation.

## 6 DISCUSSION

MDT is an emerging method for eliciting patient preferences for the benefits and harms of medicines. In the current article, we provided a complete description of the method and proposed an improved estimation procedure based on SML. The results of our computational experiments suggest that the proposed SML procedure can produce reasonable estimates of Dirichlet distributed attribute weights already with a single bisection step per trade‐off after the ranking phase, allowing MDT to be applied with smaller sample sizes or with fewer pairwise comparison questions per trade‐off compared to the more naïve centroid‐based approach that was applied in previous applications of MDT. While we presented the SML procedure in the context of the estimation of a homogeneous Dirichlet model for the attribute weights distribution, the effects of patient characteristics can readily be incorporated by specifying a Dirichlet regression model in which the distributional parameters are expressed as a function of covariates.^10^ Also, the proposed SML procedure can be applied with any preference elicitation method that results in a set of linear inequality constraints on the values of the attribute weights. Its use is therefore not restricted to MDT as described in this article.

Patient preference information is one of the main inputs for a quantitative benefit‐risk assessment, where it is combined with treatment performance data to rank the available treatment options from most to least desirable.^13^ One of such methods is stochastic multi‐criteria acceptability analysis (SMAA), in which for each treatment under consideration a set of rank acceptability indices is calculated that indicate the frequency at which that treatment is placed at each of the possible ranks between worst and best.^14^ These indices are estimated by repeatedly sampling attribute weights and treatment performance data from their respective probability distributions, which for the attribute weights requires specification of a multivariate probability distribution over the unit simplex. In practical applications of SMAA, the Dirichlet distribution is often used for this purpose, which contains as a special case the uniform distribution that was previously suggested in situations where imprecise preference information was elicited from a single decision maker.^15^ To prevent suboptimal decision making, it is important to reduce as much as possible any biases in the estimation of the parameters of the Dirichlet distribution. Our simulation results suggest that for stakeholder preferences elicited through MDT, this can be achieved by applying the SML procedure proposed in this article. With the previously applied centroid‐based approach, a larger degree of bias can be expected, especially for larger problem instances in which, to keep participant burden manageable, having more than two bisection steps per trade‐off is often not practically feasible.

Our previous work^9^ suggested that MDT with a Dirichlet population model is likely to have less than half of the sample size requirements of similarly complex discrete‐choice experiments (DCEs), the most used method in health preference elicitation.^3^ This gap in sample size requirements is expected to widen with the new SML procedure, which clearly outperformed the naive centroid‐based approach in the computational experiments. However, MDT is a less flexible preference elicitation method than the DCE as the use of thresholding requires the attributes to be measured on an ordinal or numeric scale. Because of this, MDT does not easily allow incorporation of nominal attributes with levels that are not naturally preference‐ordered, such as different modes of treatment administration. Moreover, in situations where the partial value functions cannot be assumed to be linear, additional elicitation questions are required to fully specify the additive value function.

Different variants of MDT can be envisioned depending on how the trade‐offs to evaluate with the thresholding technique are selected. In this article, we based this selection on an initial ranking of the attribute weights obtained through a swing ranking procedure. A downside of this approach is that the response tasks in swing ranking are cognitively more demanding than the pairwise comparison questions used in the thresholding phase. An alternative approach would be to make MDT fully choice‐based by eliciting the attribute ranking through a choice‐based sorting algorithm. Choice‐based sorting is however less efficient than swing ranking, meaning that a larger number of elicitation questions would be required to determine the attribute ranking. By switching from centroid‐based estimation to SML estimation, such an approach may nevertheless still be practically feasible given that the increase in the number of elicitation questions in the ranking phase can be partially offset by a decrease in the number of elicitation questions in the thresholding phase. Other approaches to adaptively generate pairwise comparison questions for attribute weights elicitation, such as entropy‐based approaches that seek to maximize the information gained from each choice question,^16^ may however be more efficient in that situation. How the different variants of MDT compare to an entropy‐based approach is an interesting avenue for further research.

## FUNDING INFORMATION

This work has received no external funding.

## CONFLICT OF INTEREST

The views presented here are those of the authors and not of their organizations.

## Data Availability

Accompanying R code is available at http://doi.org/10.5281/zenodo.5017942. The repository contains the following items: (i) an R script for generating the feasible weight spaces resulting from applying MDT with a set number of bisection steps per trade‐off (MDT_algorithm.R), (ii) an R implementation of the proposed SML procedure (SML_procedure.R), (iii) an R script for reproducing the results of our computational experiments (computational_experiments.R), and (iv) and R script for visualizing the results of the computational experiments (visualize_results.R).
